# Lameness Is Not an Excuse Anymore

**DOI:** 10.3390/ani15010068

**Published:** 2024-12-31

**Authors:** Menno Holzhauer

**Affiliations:** Ruminant Health Department, Royal GD Animal Health, P.O. Box 9, 7400 AA Deventer, The Netherlands; m.holzhauer@gdanimalhealth.com

It is an honor for me to serve as Guest Editor for this Special Issue of Animals: “Foot and Claw Health in Dairy Cows”. Trained by the late Egbert Toussaint Raven (founder of the basic steps of regular preventive claw trimming) and starting as a bovine practitioner in 1984, I have always been interested in treating lame cows, although it was difficult and sometimes dangerous work. For the past 25 years, I have focused most of my professional career on bovine lameness.

Already as a practitioner, I experienced the positive effect of gluing a block under the sound claws for several weeks. Looking back at the applied treatments during that period, it might have been better to perform claw trimming more frequently and refer to the better-equipped claw trimmers. Also, it could have been better to perform better pain management by, e.g., using more painkillers to stimulate rapid and complete recovery. I have also often thought of how much more I might have enjoyed working with lameness had I better understood its pathogenesis and better ways of treating lameness in dairy cattle.

A lot has changed since that time. I credit much of my renewed enthusiasm to my late father (veterinarian at Royal GD Animal Health), the late Dr. Dirk Peterse (Royal GD Animal Health), and the late Jan van Amerongen (Veterinary Faculty in Utrecht), and colleagues and claw trimmers both from inside and outside the country. I met inspiring colleagues at, e.g., the Lameness in Ruminants conferences from almost all over the world. Knowing each other’s faces and enthusiasm meant a lot to me; it stimulated me to work together and share knowledge for the benefit of solving worldwide problems.

Lameness is the manifestation of pain associated with a disease or injury to mainly the hind feet. Studies on the prevalence of lameness indicate that on average, more than fifty percent of the animals in Western Europe are affected. Without a doubt, lameness is the most important health and welfare issue in cattle. The urgency increased to solve fatigued cattle syndrome and serious problems with non-healing claw horn lesions secondarily infected by digital dermatitis-causing treponema bacteria. Moreover, unlike many disorders for which therapeutic interventions provide prompt relief, the disability and pain associated with lameness often linger for weeks despite effective treatment. Few disorders rival lameness in terms of costs or impact on the welfare of the affected cows. Based on the work of colleagues from Wageningen University [1,2] average expenses associated with clinical lameness conservatively approach USD 5000–10,000/year for a 100-cow herd, surpassing nearly all the other disease conditions, including mastitis and decreased fertility.

As illustrated above, lameness is a huge issue; researchers from all over the world have produced more than 5000 scientific papers and over 500 review papers on this topic (www.sciencedirect.com/; lameness dairy cows). The greatest challenge is determining what information is likely to be most critical to the day-to-day work of veterinary practitioners. Veterinarians play a key role as advisors, and many provide specific treatment to lame cows. It is important to provide practical information to assist practitioners in solving herd problems, and at the same time, address the need for scientific information on foot care and treatment practices. For example, the currently applicable regulatory legislation treatment options for organic dairy are quite different compared to those in conventional dairy systems in different countries. Similarly, pain management is a fundamental component of all therapeutic regimens, particularly in the case of lameness. Therefore, a discussion of good pain assessment and management must, nowadays, be a part of the job for the farmers and their advisors as herd verterinarian, claw trimmers and nutritionists in all animals with any kind of lameness.

A total of five articles were published in this Special Issue. The empirical papers include original research across a broad range of topics, disciplines, and countries. As such, a paper (Pyzik et al.) from colleagues from Poland evaluated the antibacterial effectiveness of bacteriophages specific for *Staphylococcus* spp. isolated from 49 dairy cows with limb and hoof infections. Moreover, a paper (Pirkkalainen et al.) from Finland studied the local and systemic inflammatory responses of 104 cows with different digital dermatitis lesions and compared macroscopical and histological findings. Along with that, a paper (Fürmann et al.) from Switzerland investigated the estimation of the prevalence of painful lesions of the digits (“alarm” lesions) in Swiss dairy herds and cow–calf operations over a three-year study period. Furthermore, a review paper (Urban-Chmiel et al.) from Poland and Slovakia conducted an analysis of the health problems of lameness in dairy cows by assessing the health and economic losses, and a paper (Holzhauer et al.) from the Netherlands reported the good results of a clinical study whereby 40 cows in 13 herds with serious claw horn disorders received a topical treatment in combination with a parenteral injection with tilmicosin.

To conclude, lameness in dairy cattle needs our full attention and the dissemination of scientific information in, e.g., scientific journals. Therefore, lameness cannot be used any longer as an excuse for other disorders like decreased fertility, hock lesions, premature cull, etc., while the knowledge is available for treatment and control. I hope that our readers find the information in papers related to the subject of lameness or claw disorders in dairy cattle, which contributes to their day-to-day work as veterinary practitioners. I want to express my sincere gratitude to all who answered the call to author or co-author an article. I also want to acknowledge Ms. Latonia Wang and the Editorial team of *Animals* for their never-ending support. Finally, I want to thank my colleagues at Royal GD Animal Health, my girlfriend, and both my sons and daughters-in-law (partly young practitioners) for the good discussions, functioning as a sounding board, and endless support.

## References

[1] Bruijnis M.R.N., Hogeveen H., Stassen E.N. (2010). Assessing economic consequences of foot disorders in dairy cattle using a dynamic stochastic simulation model. J. Dairy Sci..

[2] Edwardes F., van der Voort M., Halasa T., Holzhauer M., Hogeveen H. (2022). Simulating the mechanics behind sub-optimal mobility and the associated economic losses in dairy production. Prev. Vet. Med..

